# Keratinized Gingiva Determines a Homeostatic Behavior of Gingival Sulcus through Transudation of Gingival Crevice Fluid

**DOI:** 10.1155/2011/953135

**Published:** 2011-11-15

**Authors:** Maria Luiza P. Lagos, Adriana Campos Passanezi Sant'Ana, Sebastião Luiz Aguiar Greghi, Euloir Passanezi

**Affiliations:** ^1^MINTER, State University of Londrina and School of Dentistry at Bauru, University of São Paulo, Al. Dr. Octávio Pinheiro Brisolla 9-75, 17012-912 Bauru, SP, Brazil; ^2^School of Dentistry at Bauru, University of São Paulo, Al. Dr. Octávio Pinheiro Brisolla 9-75, 17012-912 Bauru, SP, Brazil

## Abstract

*Objective*. To shed light on the role of KG, its influence on periodontal behavior was investigated. *Methods*. Tissue fluid transudation was assessed in alveolar mucosa (P1A), outer gingival margin (P1B), at entrance of (P2) and within gingival sulcus (P3), before and after chewing of fibrous food in 16 patients portraying ≥2 mm KG at one tooth (group 1), and <2 mm at another homologous tooth (group 2). *Results*. There was a significant increase in GCF after chewing at P1B and P3 in group 1 and at P1A in group 2 (*t*-test, *P* < 0.05). *Conclusions*. The results suggest that KG plays a role in marginal periodontal homeostasis.

## 1. Introduction

Since many years, a great number of studies have been discussing the role of keratinized gingiva (KG) in marginal periodontal behavior, either accepting that a minimum of 2.0 mm of KG width is required to maintain marginal periodontal health [1–6] or suggesting that KG width is negligible if excellent oral hygiene is performed [7–15]. 

Areas with narrow width of KG seem to be prone to periodontal attachment loss [7], which could result in recession of the gingival margin in the presence of risk factors. Lang and Löe [1] have demonstrated that such areas show clinical signs of inflammation even in the absence of dental plaque, as evidenced by an increase in gingival crevicular fluid (GCF) flow rate, which suggests that tissue behavior is mainly associated to pathological more than to physiological processes under these conditions [5].

An increased GCF flow rate from gingival sulcus is an early sign of clinical inflammation [16–20]. In the absence of dental plaque, the increase of GCF flow rate in areas presenting <2.0 mm of KG width could be influenced by a close proximity of the dentogingival unit to the alveolar mucosa, which is more permeable and mobile to allow primary defense against microorganisms and its products [5, 17]. Some factors can explain the increase in GCF flow rate, including (i) increase in tissue hydrostatic pressure close to junctional epithelium induced by bacterial plaque accumulation [18, 19, 21]; (ii) mobility of gingival margin and increase in marginal blood flow caused by mechanical stimulus from tooth mobility, frenum pull, toothbrushing, and chewing [22]; (iii) histamine intravenous injection or development of inflammation [23]. These findings suggest that chemical or mechanical irritation is necessary to the production of GCF [21, 24]. 

GCF is considered to develop an important protective role on the defense mechanisms of gingival sulcus through the presence of defensive substance, such as PMN-neutrophils, and through mechanical properties, such as the flushing action, capable of removing carbon particles and bacteria from the gingival sulcus [20, 24–28]. Taken together, these findings suggest that the absence of GCF in the absence of mechanical stimulation would represent gingival health, while its presence in the absence of mechanical stimulation would represent gingival inflammation [16]. 

To the best of our knowledge, no study has investigated if there are differences in GCF flow rate in areas with a narrow or wide width of keratinized gingiva, aiming at determining if a wider width of keratinized gingiva would allow a better dissipation of GCF. Considering so, the aim of this study was to investigate the role of KG gingiva in the homeostasis of gingival margin by evaluating GCF flow rate in healthy sites before and after a mechanical stimulus.

## 2. Material and Methods

This study was approved by the Committee of Ethics in Research of School of Dentistry at Bauru-USP (no. 10/2002). The sample was selected according to the following inclusion criteria: presence of a buccal healthy site showing ≥2.0 mm width of KG at bicuspids or molars and a homologous site presenting <2.0 mm of KG; good systemic health; optimal oral hygiene status. It was excluded from the study pregnant or lactating women, patients prescribed with restricted drugs or antibiotics in the 6 and 3 months previous to data collection, those taking medicines capable of inducing gingival hyperplasia (e.g., calcium channel blockers, cyclosporine, and anticonvulsants), smokers, drugs, or alcohol abusers.

### 2.1. Sample Size

**Figure 1:**
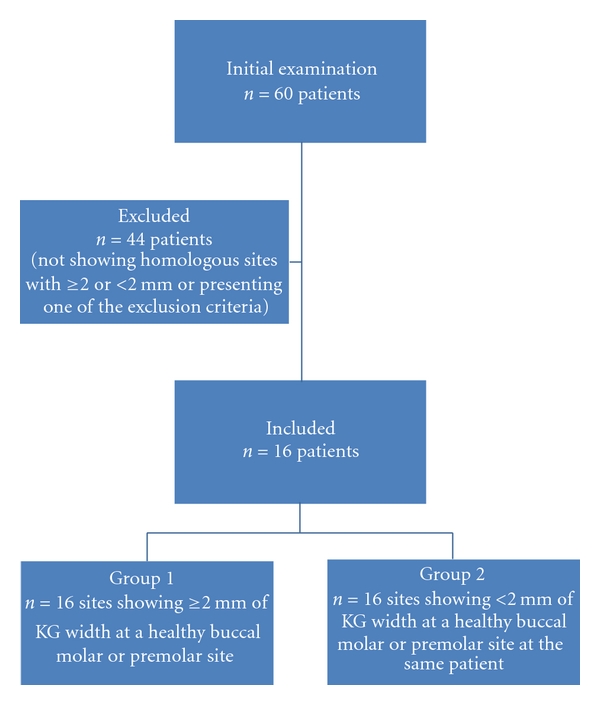
Flow diagram of sample selection and study design.

A total of 60 patients were initially examined, but only 16 were found in accordance to inclusion and exclusion criteria (Figure 1). Group 1 was composed of 16 strictly healthy premolar and molar buccal sites showing ≥2 mm of KG width, and group 2 was composed of 16 strictly healthy premolar or molar buccal sites showing <2 mm of KG width in the same patients, in a split-mouth design.

### 2.2. Standardization Procedures

**Figure 2:**
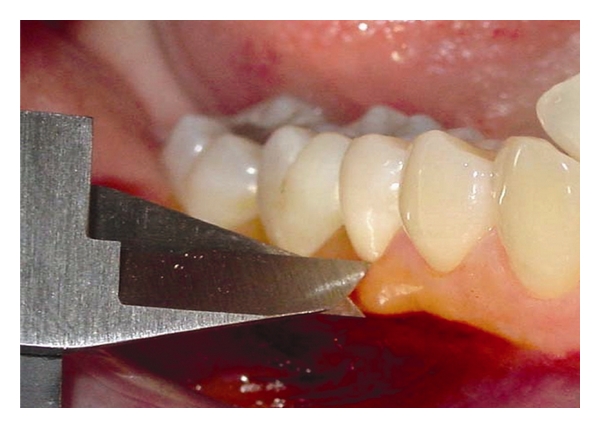
Measurement of KG width with digital caliper after staining of the gingiva with Schiller's solution.

In order to assure the absence of gingival inflammation, all patients were submitted to scaling and root planning and oral hygiene instruction before data collection. The reliability of these conditions was assessed by the evaluation of plaque index [29], sulcular bleeding index [30], and probing depth, measured with a millimeter periodontal probe (HuFriedy, Chicago, USA). KG width was measured by a digital caliper (727 ME, WIT, Brazil) as the distance from gingival margin to mucogingival junction at buccal sites of the selected teeth, after staining of the tissues with a Schiller solution (Figure 2). All clinical examinations and procedures were under the responsibility of a single-trained examiner. Clinical measurements were performed 24–72 hours before GCF collection to avoid any interference on the gingival sulcus physiology.

### 2.3. GCF Collection and Quantitation

**Figure 3:**
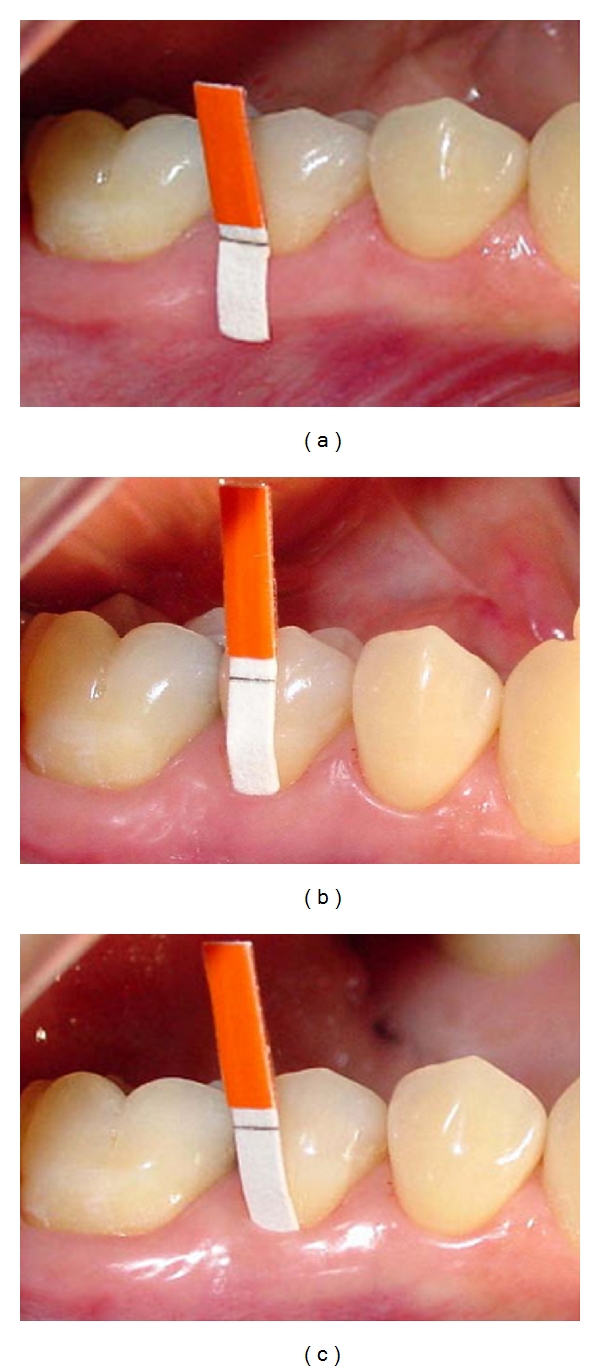
(a) Positioning of paper strip overlying the tooth, gingiva, and alveolar mucosa for obtaining GCF at P1A (alveolar mucosa) and P1B (gingival margin); (b) positioning of paper strip at gingival margin without penetrating gingival sulcus-P2; (c) positioning of paper strip into gingival sulcus-P3.

The collection of GCF was accomplished by imbibing of PerioPaper strips (Oralflow, NY, USA) as proposed by Löee and Holm-Pedersen [16]. After preventing the contact of the tooth by the tongue, GCF was sampled according to extra- and intracrevicular methods. In the former, the paper strips were closely fitted over the tooth crown and the buccal soft tissue surface, extending up to the alveolar mucosa (Figure 3(a)), allowing the imbibing of the strips by fluid exuding through the bordering edge of alveolar mucosa (P1A) and through the gingival margin (P1B). GCF was also collected at superficial position (P2), at the entrance of gingival crevice (Figure 3(b)), and deep intracrevicular position (P3), in which the strip was introduced to the base of gingival crevice (Figure 3(c)), as determined by minimal tactile resistance upon its introduction [22, 25]. Paper strips were kept in position for 60 seconds, allowing the imbibing of paper strips in a standardized period, as reported before [31, 32] and supported by the results obtained in a pilot study involving 3 patients (data not shown). 

GCF was collected in two different moments: before and after chewing a fresh fibrous food meal (cooked bovine steak) for 10 minutes. Collection of GCF before chewing was performed 24–72 hs after clinical measurements, in a rested position. After 24 hours, patients were recalled and eat the cooked fibrous meal during 10 minutes. After that, collection of GCF according to the described methodology was performed. 

**Figure 4:**
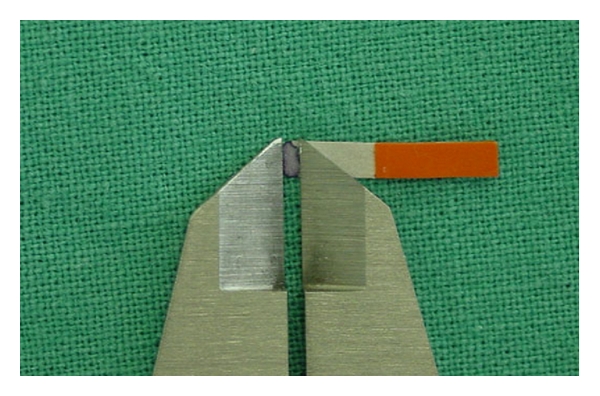
Linear measurement of paper strip area stained by ninhidrine solution. Note that the digital caliper was positioned in such a way to allow the measurement of the greatest linear stained area.

The strips were then heat air dried and imbibed in a 2% alcoholic solution of ninhydrine [17], which provides comparable results to electronic devices [33] and again heat air dried. The stained areas were linearly measured by a digital caliper (Figure 4). Measurements were obtained in inches and converted to millimeters for statistical analysis. 

### 2.4. Statistical Analysis 

The data was statistically evaluated in a statistical program for Windows (SigmaStat). Comparisons between groups before and after chewing stimuli were analyzed by unpaired *t-*test. Intragroup comparison of GCF collection before and after chewing stimuli was analyzed by paired *t-*test. A 95% confidence level was established for all statistical analysis (*α* = 0.05).

## 3. Results

Periodontal status of test and control sites before collection of GCF is shown at Table 1. No differences were found between groups in probing depth, plaque index, and bleeding index. Significant differences were observed between groups only in KG width (*P* < 0.05). 

The amount of GCF collected from groups 1 and 2 sites before and after chewing stimuli is described in Table 2. Significant differences between groups were found at P1A before mastication stimulus and at P2 and P3 after mastication stimulus. Intragroup analysis by paired *t-*test showed significant increases in GCF at P1B and P3 in group 1 and at P1A in Group 2.

## 4. Discussion

The results obtained in this study have shown that tissue fluid transudates rather through gingival sulcus than through alveolar mucosa in areas presenting at least a 2 mm KG width, while areas presenting lesser than 2 mm of KG width show transudation of tissue fluid by alveolar mucosa. These findings suggest that the protective role [16, 21, 24–28] exerted by GCF is compromised in areas with a narrow width of KG. 

This study is important because no previous reports have evaluated the role of KG in the homeostatic response of marginal periodontal under strictly healthy and physiological conditions. Miyasato, Crigger, and Egelberg [34] also evaluated the homeostatic response of 16 dental students or members of the Dentistry Faculty showing “appreciable” (≥2 mm) or “minimal” amount of KG (<1 mm) in plaque-free contralateral or unilateral sites and showed that both areas showed minimal amounts of GCF in a resting state, as observed in the present study. When plaque was allowed to accumulate, a significant increase in GCF flow rate was observed for both groups, with no differences between groups, as gingival inflammation developed, but no mechanical stimulus was performed to induce GCF flow rate. 

GCF flow rate is usually a measure of gingival inflammation, since its exudation increases in the presence of inflammation and either is not present or present in small quantities in healthy situations [16, 19–21, 28, 29, 34]. Indeed, the volume or resting GF increases as periodontal pockets develop [20, 34, 35]. While in healthy sites GCF represents a transudate of interstitial tissues, in the course of gingivitis and periodontitis, it is transformed into a true inflammatory exudate [19, 20]. In this context, the relevance of GCF in the control of a subgingival microbiota compatible with periodontal health should be emphasized [17, 20, 25]. 

No differences between groups were observed at gingival margin (P1B) and superficial (P2) and deep (P3) intracrevicular positions before chewing stimuli. This can be possibly explained by the fact that in clinically healthy conditions the amount of GCF transudation through gingival sulcus is minimal [16, 19–21, 28, 29, 34], which might account for differences between the present study and others published in literature [1, 16]. However, a significant difference between groups was found at P1A (alveolar mucosa edge), indicating that, in a resting position, dissipation of tissue fluid occurs mainly by alveolar mucosa, which is more permeable and mobile to allow metabolic interchange. 

This result also seems to indicate that some tissue fluid from alveolar mucosa can dissipate toward the gingival sulcus in narrow KG, suggesting that the greater the distance from gingival sulcus to alveolar mucosa, the lesser the influence of alveolar mucosa on the physiological behavior of gingival sulcus. According to Siegel [36], the identification of an increased flushing of tissue fluid through the epithelial barrier of the alveolar mucosa is highly suggestive of the permeability of this tissue. 

After mechanical stimuli provided by chewing a fibrous meal, a significant increase in transudation of GCF through gingival sulcus was observed at P1B and P3 positions for sites with ≥2 mm width of KG, with a trend to increase also at P2, although not statistically significant. The same stimulus resulted in increase in transudation of tissue fluid trough alveolar mucosa (P1A), while the amount of GCF collected from gingival sulcus (P1B, P2 and P3) suffered minor not significant variations in sites showing <2 mm of KG width. These results are in agreement with other studies [16–18, 20, 22, 25] showing that mechanical stimulus is capable of increasing GCF flow rate even in healthy areas.

These findings also indicate a more pronounced permeability of alveolar mucosa than KG, which allows the crossover of substances from the inner to the outer environment and *vice versa* [36]. This might be relevant in the control of periodontal homeostatic response through an early recognition of potentially aggressive bacterial antigens. The increased fluid rate observed at P1A position implies in increased alveolar mucosa transudation influx, probably related to the tissue blood supply necessary to accomplish the metabolic demands for functional tissue mobility [5].

Besides, the increase in GCF flow rate at gingival sulcus in group 1 reflects the role of GCF in flushing the gingival sulcus, corroborating the results obtained by previous studies [16, 17, 20, 25, 26]. This protective role was not observed in group 2, since GCF flow rate remained the same after chewing, suggesting that a wider area of KG is more compatible with marginal periodontium homeostasis, playing a regulatory role on the control of concentration and distribution of GCF flow rate [16, 17, 22, 25, 37, 38]. These results might explain why areas showing ≥2 mm of KG width are more compatible with marginal periodontal health than areas <2 mm wide, which requires a strict maintenance program in order to prevent plaque accumulation [1–8, 11, 14].

Kennedy et al. [14] found that gingival health could be maintained in patients under professional plaque control but not in those without professional care in areas presenting an inadequate KG width. In their study, patients previously submitted to free gingival autografts procedures that did not participate in the supportive periodontal treatment for a 5-year period have not shown significant gingival inflammation and/or recession overtime, contrariwise to those portraying inadequate KG. From these results, it can be assumed, as in the present study, that the existence of an adequate KG width is a natural requirement to allow a reliable homeostatic response of the marginal periodontium when the patient performs personal routine dental plaque control. 

In the absence of dental plaque, the increase in flow rate of GCF in areas <2 mm in width could be influenced by a close contact between the dentogingival unit and the alveolar mucosa, which is more permeable and mobile, thus allowing primary defense mechanisms to be activated against bacterial challenge. Additionally, the increase in tissue hydrostatic pressure close to the junctional epithelium may contribute to a more pronounced flow rate of GCF in narrower areas [18–21, 39, 40]. Since the attached gingiva is a fraction of keratinized gingiva and extends up to the boundaries of the mucogingival junction, these areas must be capable of neutralizing the tension transmitted by the alveolar mucosa under muscle action in such a way that marginal gingival mobility is prevented [5].

This study also showed that the mastication of natural fibrous food produces alterations in GCF flow rate which can vary according to the width of KG. This behavior could be engaged to the dental mobility induced by chewing, that would transmit a stimulus to gingival blood vessel walls through oxytalan fibers [41–45], giving rise to an increased blood vessel transudation, which in turn would be related to the changes in GCF flow rate [18, 19]. Additionally, the mobilization of gingiva can also be attributed to food excursion and/or functional demands of the alveolar mucosa, since both inadequate and adequate KG groups experienced significant variation in GCF flow rate, more pronounced in areas presenting an adequate width of KG.

The main limitation of this study is the small number of subjects included in the study, due to difficulties in finding patients who presented homologous sites with ≥ or <2 mm of KG width and did not present any of the exclusion criteria. This might explain why no significant differences were found at P2 position before and after chewing in group 1, since an increase in GCF was noticed. By the other side, this was a split-mouth design study, which implies that “test” and “control” sites were in the same patients. All patients were submitted to professional plaque control by scaling and root planning and prophylaxis, and oral hygiene instruction in order to assure that all patients were clinically healthy at the moment of GCF collection.

These results suggest that KG plays a definite role in controlling the physiology of gingival sulcus, allowing the transudation of GCF through the gingival sulcus, resulting in an adequate protective role essential to the maintenance of gingival health and periodontal homeostasis.

## 5. Conclusions

The results obtained in this study suggest that a wider area of keratinized gingiva favors physiological behavior of the gingival sulcus by a better dissipation of GCF; a closer proximity of gingival margin and alveolar mucosa influences the dissipation of tissue fluid through alveolar mucosa, which is more permeable and mobile, impairing primary defense of gingival sulcus by the concentration of GCF. 

## Figures and Tables

**Table 1:** Baseline sample characterization according to plaque index (PI), sulcular bleeding index (SBI), probing depth (PD), and keratinized gingiva (KG) width for sites included in groups 1 (≥2 mm KG) and 2 (<2 mm KG).

Clinical parameter	Group 1			Group 2		
	*n*	*x*	sd	*n*	*x*	sd
PI	16	0.000	0.000	16	0.000	0.000
SBI	16	0.000	0.000	16	0.000	0.000
PD	16	0.875	0.223	16	0.750	0.258
KG	16	2.912*	0.717	16	0.993*	0.525

*P < 0.05; parametric *t*-test.

**Table 2:** Parametric *t*-test for intergroups analysis of gingival fluid flow rate according to the different collection methods before mastication stimulus.

Position	Group 1 (*n* = 16)			Group 2 (*n* = 16)		
	Before	After	*P*	Before	After	*P*
P1A	0.82 ± 0.79^a^	0.72 ± 0.64^A^	0.54	0.33 ± 0.38^b^	0.68 ± 0.79^A^	0.04
P1B	0.48 ± 0.72^a^	0.89 ± 0.69^A^	0.03	0.65 ± 0.57^a^	0.55 ± 0.57^A^	0.58
P2	0.59 ± 0.36^a^	0.88 ± 0.62^A^	0.06	0.46 ± 0.35^a^	0.50 ± 0.38^B^	0.66
P3	1.05 ± 0.45^a^	1.38 ± 0.69^A^	0.04	0.96 ± 0.39^a^	0.95 ± 0.24^B^	0.91

Equal lowercase letters mean no significant differences between groups before mastication stimulus (*P* ≥ 0.05); different lowercase letters mean significant differences between groups before mastication stimulus (*P* < 0.05); equal capital letters mean no significant differences between groups after mastication stimulus (*P* ≥ 0.05); different capital letters mean significant differences between groups after mastication stimulus (*P* < 0.05).
